# Public preferences and attitudes towards the disclosure of medical errors: a survey in Iran

**Published:** 2018-08-04

**Authors:** Akram Heidari, Masoomeh Razaghi, Fariba Asghari

**Affiliations:** 1 *Assistant Professor, Spiritual Health Research Center, Qom University of Medical Sciences, Qom, Iran.*; 2 *Researcher, Spiritual Health Research Center, Qom University of Medical Sciences, Qom, Iran. *; 3 *Associate Professor, Medical Ethics and History of Medicine Research Center, Tehran University of Medical Sciences, Tehran, Iran.*

**Keywords:** *Medical error*, *Disclosure*, *Consumer preferences*

## Abstract

Awareness of the occurrence of medical errors is the right of patients and duty of the health service providers. This study was conducted to evaluate to what extent people want to know the occurrence of an error in their medical care, what they expect to be disclosed about medical error, and what are the influential factors in filing a lawsuit against physicians in disclosed medical errors from their point of view.

In this cross-sectional survey, 1062 people residing in the city of Qom, Iran, were telephone interviewed using the random digit dialing method. The questionnaire used consisted of 4 demographic questions and 2 scenarios of major and minor medical error; the participants were asked if the physician should disclose the error in each scenario. The questionnaire also consisted of 16 questions about other issues related to error disclosure. Data were analyzed through descriptive and inferential statistics in SPSS software.

About 99.1% of the study population believed that errors had to be disclosed to patients. They all wished to know that measures would be taken to prevent further errors. Moreover, 93.1% of the participants expected an explanation on the incident. As for the factors that decreased the likelihood of taking legal action against the physician from the viewpoint of the study population, treatment of the complications (96.1%) and honesty of the physician (95.8%) had the highest frequency.

Based on the considerable preference of patients for error disclosure, it is recommended that physicians disclose all minor and major errors sympathetically and with transparency, honesty, and efforts to prevent future errors.

## Introduction

Medical errors jeopardize the safety of patients (1). Evidence suggests that medical errors are of paramount significance in the quality control of health care (2, 3). In line with patient autonomy, awareness of the occurrence of medical errors is the right of patients and a duty of health service providers. Lack of error disclosure is patient deception and results in mistrust to medical staff. Regarding error disclosure, there is a considerable gap between what the patients expect and what physicians do (4). Studies have shown that people have a great desire to learn about medical errors (5-8). In a study by Mazor et al., 98.8% of the participants wished to learn about medical errors (9); Hobgood et al. reported this rate as 99% (10) and 76% (11) in their studies. This rate was 89% in the study by Ushie et al. (8). In the study by Matlow et al., all parents stated that they would like to be informed of the error and 77% of them reported that they would also like their child to be informed (6). 

Despite the great desire of the patients to have information on medical errors, physicians are not very interested in error disclosure (12-15). A study by Ghalandarpoorattar et al. showed that only 16.7% of the studied surgeons had admitted their last medical error to their patients (16). In a study by Loren et al., only 53% of pediatricians and pediatric fellows declared that they would definitely admit their mistakes to patients (17).

Physicians have different reasons for avoiding error disclosure. According to studies, the most important obstacles to error disclosure‎ are legal complaints by patients, concerns regarding patients’ trust, fear of losing their professional reputation, and inadequate education (16, 18, 19).

Since one of the greatest concerns of physicians regarding error disclosure is legal complaints of the patients, it is necessary to evaluate if error disclosure is correlated with legal complaints against physicians, which factors are related to higher likelihood of a formal complaint by patients, and the information that patients wish to acquire when an error occurs in order to maintain their trust and confidence in their physician. 

This study was conducted to evaluate to what extent people want to know the occurrence of an error in their medical care, what they expect to be disclosed about medical errors, and what factors are related to filing a lawsuit against physicians in disclosed medical errors. 

## Methods

This study was a cross-sectional survey. The study population consisted of individuals residing in the city of Qom, Iran. The sample size was estimated at 1024 individuals for α = 0.05, *P* = 0.60, and d = 0.03. Thus, 1062 individuals were entered into the study. Sampling was performed using the random digit dialing method. An Internet software program was used to list random telephone numbers (randomnumbergenerator.intemodino.com/en/generate-random-numbers.html). The inclusion criteria consisted of being older than 18 years of age, residency in Qom, complete consciousness, and the ability and willingness to answer questions. For the random selection of the interviewees from among the family members, the person whose birthday was closer to the interview date was selected since this variable is independent of attitude and unrelated to other demographic variables such as gender and age (20, 21). All telephone interviews were conducted by one person between 6-9 pm. An average of 10 minutes was dedicated to each interview for every person (30 to 40 interviews per week). At the beginning of each interview, the operator introduced herself to the participants, explained the objectives of the project, and obtained an oral consent of participation to continue the study. The telephone numbers of non-residential places were excluded. The interviews were conducted from June 2013 to January 2014. 

The questionnaire used in this study contained 4 demographic questions (age, sex, educational level, and marital status), 1 question about whether the participants, their family members, or relatives had experienced any medical errors, 2 scenarios, and 17 questions about other issues related to error disclosure‎. The first scenario was constructed on a minor medical error (with limited and controlled adverse effects on the patients) and the second on a major medical error [which resulted in injury and intensive care unit (ICU) admission], and the participants were asked whether the physician should disclose the error to patients in each situation (Table 1). 

**Table 1:** The two scenarios suggested in the questionnaire

Minor Error Scenario: You are admitted to the hospital. The physician prescribes insulin for elevated blood glucose levels, but makes a mistake in the prescribed dose. Due to receiving excess insulin, your blood sugar drops and you faint. The physician administers an intravenous sugar solution and you feel better after one hour.
Major Error Scenario: You are hospitalized. You go into shock due to the injection of the wrong medicine and require a few extra days of ICU admission after resuscitation.

In addition, 14 questions were asked on the attitude of the patients; 7 questions were related to components of the expected information when disclosing an error and 7 questions addressed the factors that increased or decreased the likelihood of pressing charges against the physician. Moreover, 3 questions were asked to evaluate the possibility of pardoning or taking legal action against the physician in case of the self-disclosure of the medical error, possibility of terminating the physician-patient relationship in case of self-disclosure, and the possibility of pardoning or taking legal action against the physician in case of error disclosure by a colleague. 

The questionnaire was designed by the authors based on a review of the relevant literatures. The psychometrics of the questions was reviewed by 3 medical ethics experts using a qualitative method. The face and content validity of the questions and the comprehensiveness of the questionnaire for the study objectives were confirmed after a few revisions. To enhance the clarity of the questions, a pilot study was conducted on 10 individuals. The reliability of the questionnaire was evaluated using the test-retest method on 30 participants with a 1-week interval. The Cohen's kappa coefficient of the questions ranged between 0.87 and 1. 

The data were analyzed using descriptive (frequency, mean, and standard deviation) and inferential statistics (Pearson coefficient, chi-square test, Fisher's exact test, and Odds Ratio) in SPSS software (version 20, IBM Corporation, Armonk, NY, USA). All *P* values < 0.05 were considered significance.

This study was approved by the Ethics Committee of Qom University of Medical Sciences, Iran. 

## Results

Of the 1327 individuals contacted through telephone calls, 1062 individuals participated in the study (RR = 80%), among which 786 (74.2%) were women. The mean age of the study population was 35.20 ± 11.24 years (range: 18-75 years) (Table 2). Among the study population, 229 (21.6%) stated that they, their family members, or their relatives had experienced medical errors. 

Among the study population, 99.1% expressed their preferences for error disclosure; 962 (90.6%) and 1055 individuals (99.5%) reported their preferences for error disclosure in the minor error scenario and the major error scenario, respectively. Table 3 presents the required components of medical error disclosure and table 4 shows the likelihood of taking legal action against physicians in case of error disclosure from the people’s point of view. The results showed that error disclosure by the physician compared to error disclosure by another physician had lower possibility of formal complaints (OR = 2.87, CI = 2.22-3.70) (*P* < 0.0001).

**Table 2:** The frequency of demographic variables in the study population

Variables		Number	%
Age groups (year)	≤ 20	51	4.8
	21-30	394	37.1
	31-40	345	32.5
	41-50	161	15.2
	51-60	83	7.8
	> 60	27	2.5
	Total	1061	100
Education	Illiterate	36	3.4
	Reading and Writing	14	1.3
	Elementary school	199	18.8
	Middle school	162	15.3
	High school	365	34.5
	Academic education	283	26.7
	Total	1059	100
Marital status	Married	893	84.2
	Single	143	13.5
	Divorced	2	0.2
	Widowed	22	2.1
	Total	1060	100

**Table 3:** The required components of medical error disclosure

**Component**	**Yes** **N (%)**	**No** **N (%)**	**Total** **N (%)**
Measures to prevent further errors	1050 (99.7)	3 (0.3)	1053 (100)
Disclosing the cause of error	1042 (98.1)	20 (1.9)	1062 (100)
Disclosing what exactly happened	989 (93.1)	73 (6.9)	1062 (100)
Compensation for injury	745 (70.2)	317 (29.8)	1062 (100)
Expressing regret and apology	713 (67.2)	348 (32.8)	1061 (100)

**Table 4:** The frequency of the likelihood of taking legal action against physicians in case of medical error disclosure from the viewpoint of the study population

**Condition**	**Forgive** **N (%)**	**Legal ** **action** **N (%)**	**It ** **depends** **N (%)**	**Total** **N (%)**
Error disclosure by physician	544 (51.3)	113 (10.7)	403 (38)	1060 (100)
Error disclosure by another physician	431 (40.7)	257 (24.2)	372 (35.1)	1060 (100)

In the present study, 386 participants (36.4%) stated that they would continue their treatment course with the physician who made the error while 517 individuals (48.8%) stated that they would change their physician. The rest of the participants (n = 157, 14.8%) said that their decision depended on various factors. As for the factors that reversely correlate with the likelihood of legal action against the physician, compensation for the error and treatment of the complications and honesty had the highest frequency (Table 5).

**Table 5:** Factors affecting the likelihood of taking legal action against physicians in case of medical errors from the viewpoint of the study population

**Factor**	**Increased the likelihood ** **of taking legal action** **N (%)**	**No effect** **N (%)**	**Lowered the ** **likelihood of taking ** **legal action** **N (%)**	**Total** **N (%)**
Error compensation and treatment of complications	3 (0.3)	38 (3.6)	1010 (96.1)	1051 (100)
Honesty and truthfulness of the physician	2 (0.2)	43 (4)	1017 (95.8)	1062 (100)
Physician’s compassionate communication	1 (0.1)	242 (22.8)	818 (77.1)	1062 (100)
Physician’s apology	8 (0.8)	311 (29.2)	743 (70)	1062 (100)
Informed about error by another person	330 (31.1)	621 (58.5)	111 (10.5)	1062 (100)
High cost of treatment	782 (73.6)	242 (22.8)	38 (3.6)	1062 (100)
Severity of complications	899 (84.6)	128 (12.1)	35 (3.3)	1062 (100)

The participants who reported that they, their family members, or their relatives have experienced medical errors believed that the physician should undertake error disclosure about minor medical errors significantly more than other participants (94.8% versus 89.4%) (*P* < 0.05). The effect of the history of exposure to medical error on the attitude of the participants regarding the factors changing the likelihood of taking legal action from their point of view was only significant regarding the severity of the complication. About 90.4% of the participants with a history of exposure to medical errors believed that the severity of the complications increased the likelihood of taking legal action against the physician while 83.1% of the participants with a negative history of exposure were of this opinion (OR = 1.92, CI = 1.19 to 3.08) (*P* < 0.05).

## Discussion

Honest and complete disclosure of the medical error is a sign of the physicians’ respect for patient autonomy, respect for the patients, and effort to maintain their trust. In line with other studies, the results of the present study showed that people wish to know about the occurrence of medical errors (6, 8-11). 

The most important aspect of error disclosure to the participants, besides knowing what has happened and its cause, was that error would lead to measures to prevent further errors as it made them believe that the harm or suffering had a valuable effect on the improvement of patient care. 

The present study showed that the participants believed that people had lower tendency toward filing a complaint if the physician disclosed his/her error compared to error disclosure by another physician. However, the results revealed that the participants believed that learning about medical errors from another physician had no effect on the likelihood of legal action (Table 5), while they believed that it was the physician’s honesty that lowered its likelihood. In the study by Coffey et al., participants believed that transparency in error occurrence could rebuild trust in the patient-physician relationship (5). 

Despite the fact that physicians are concerned with legal action upon error disclosure, the results of the study showed that people had a tendency to receive the truth from the physician in a compassionate way, and honesty and error compensation decreased their tendency to take legal action. In addition to the efforts to decrease physical and mental injuries resulting from the error, people believe that the physician’s sympathy and honesty help to maintain a good relationship with the patient which lowers the likelihood of legal actions. A study by Mazor et al. also showed that full disclosure of medical error decreased the reported likelihood of changing physicians and increased the satisfaction, trust, and positive psychological responses of the patients (9). In an experimental study by Nazione and Pace, the findings showed that empathy could reduce anger and complaint rate among participants (22). 

Many studies showed that people’s views were negative toward health care providers in cases in which the error caused a severe complication and patients were not informed about the error (8, 23-25). Cleopas et al. in their study found that slow and ineffective disclosure of the error makes a negative impression on patients (26). Ushie et al. in their study showed that volunteer disclosure would significantly reduce the patient’s tendency toward taking legal action (8). 

Medical idealism does not accept medical errors. It is therefore very painful to disclose medical errors, and thus, inability to satisfy the patients’ needs. In addition, fear of legal action is one of the greatest concerns of physicians regarding error disclosure (16, 18, 19). In these circumstances, consultative and supportive services provided by the employing hospital or clinic are essential for coping with the situation and managing it appropriately according to the interests of the patients. Studies have shown that physicians lack the necessary capabilities in this regard and do not have the required self-confidence to face these situations (16). On the other hand, many factors like systemic factors are involved in medical errors and it is not therefore fair to blame it all on physicians. A systemic approach to medical errors not only helps with an appropriate and prompt disclosure of the error, but also provides the required support for the physician regarding emotional pressures and also enhances the culture of error discourse and planning for service improvement (27).

This study had some limitations. Many participants had no exposure to medical errors in the real world so the results may not be generalizable to those who have experienced medical errors personally. However, we tried to create an appropriate understanding of the situation through presenting 2 scenarios. 

The appropriate timing of the interviews in order to benefit from the presence of all family members and the good response rate were the strong points of this study; however, women comprised the majority of the participants, which decreases the generalizability of the results. According to the results of this study, people accept medical errors from physicians provided that they are disclosed honestly and sympathetically and efforts are made to prevent and avoid future errors, and hence, they are less likely to take punitive action. Based on the result of this survey and considerable preferences for error disclosure in the study population, it is recommended that physicians disclose all minor and major errors sympathetically and with transparency, honesty, and efforts to prevent and avoid future errors. The findings of the present study emphasize the importance of an honest patient-physician relationship. Patients desire an acknowledgment from their physicians of even minor errors, and doing so may actually reduce the risk of punitive actions. These findings reinforce the importance of open communication between patients and physicians.

## References

[1] Grober ED, Bohnen JM (2005). Defining medical error. Can J Surg.

[2] Brennan TA, Leape LL, Laird NM (2004). Incidence of adverse events and negligence in hospitalized patients: results of the Harvard medical practice study I.1991. Qual Saf Health Care.

[3] Forster AJ, Asmis TR, Clark HD (2004). Ottawa hospital patient safety study: incidence and timing of adverse events in patients admitted to a Canadian teaching hospital. CMAJ.

[4] Chan DK, Gallagher TH, Reznick R, Levinson W (2005). How surgeons disclose medical errors to patients: a study using standardized patients. Surgery.

[5] Coffey M, Espin S, Hahmann T (2017). Parent preferences for medical error disclosure: a qualitative study. Hosp Pediatr.

[6] Matlow AG, Moody L, Laxer R, Stevens P, Goia C, Friedman J (2010). Disclosure of medical error to parents and paediatric patients: assessment of parents' attitudes and influencing factors. Arch Dis Child.

[7] Norrish MI (2015). Disclosure of medical errors in Oman: public preferences and perceptions of current practice. Sultan Qaboos Univ MedJ.

[8] Ushie B, Salami K, Jegede A, Oyetunde M (2013). Patients’ knowledge and perceived reactions to medical errors in a tertiary health facility in Nigeria. Afr Health Sci.

[9] Mazor KM, Simon SR, Yood RA (2005). Health plan members' views about disclosure of medical errors. Am J Manag Care.

[10] Hobgood C, Tamayo-Sarver JH, Elms A, Weiner B (2005). Parental preferences for error disclosure, reporting, and legal action after medical error in the care of their children. Pediatrics.

[11] Hobgood C, Peck CR, Gilbert B, Chappell K, Zou B (2002). Medical errors—what and when: what do patients want to know?. Acad Emerg Med.

[12] Al-Nomay NS, Ashi A, Al-Hargan A, Alshalhoub A, Masuadi E (2017). Attitudes of dental professional staff and auxiliaries in Riyadh, Saudi Arabia, toward disclosure of medical errors. Saudi Dent J.

[13] Hs AS, Rashid A (2017). The intention to disclose medical errors among doctors in a referral hospital in North Malaysia. BMC Med Ethics.

[14] Bari A, Khan RA, Rathore AW (2016). Medical errors; causes, consequences, emotional response and resulting behavioral change. Pak J Med Sci.

[15] Mazor K, Roblin DW, Greene SM, Fouayzi H, Gallagher TH (2016). Primary care physicians’ willingness to disclose oncology errors involving multiple providers to patients. BMJ Qual Saf.

[16] Ghalandarpoorattar SM, Kaviani A, Asghari F (2012). Medical error disclosure: the gap between attitude and practice. Postgrad Med J.

[17] Loren DJ, Klein EJ, Garbutt J (2008). Medical error disclosure among pediatricians: choosing carefully what we might say to parents. Arch Pediatr Adolesc Med.

[18] Levinson W (2009). Disclosing medical errors to patients: a challenge for health care professionals and institutions. Patient Educ Couns.

[19] Mardani Hamooleh M, Shahraki Vahed A (2009). The obstacles in reporting nursing error: a nurses’ perspective. Medical Ethics and History of Medicine.

[20] Waksberg J (1978). Sampling methods for random digit dialing. Journal of the American Statistical Association.

[21] Lavrakas PJ (2008). Encyclopedia of Survey Research Methods.

[22] Nazione S, Pace K (2015). An experimental study of medical error explanations: do apology, empathy, corrective action, and compensation alter intentions and attitudes?. J Health Commun..

[23] Blendon RJ, DesRoches CM, Brodie M (2002). Views of practicing physicians and the public on medical errors. N Engl J Med..

[24] Schwappach DL, Koeck CM (2004). What makes an error unacceptable? A factorial survey on the disclosure of medical errors. Int J Qual Health Care..

[25] Witman AB, Park DM, Hardin SB (1996). How do patients want physicians to handle mistakes?: A survey of internal medicine patients in an academic setting. Arch Intern Med..

[26] Cleopas A, Villaveces A, Charvet A, Bovier P, Kolly V, Perneger T (2006). Patient assessments of a hypothetical medical error: effects of health outcome, disclosure, and staff responsiveness. Qual Saf Health Care..

[27] Perez B, Knych SA, Weaver SJ (2014). Understanding the barriers to physician error reporting and disclosure: a systemic approach to a systemic problem. J Patient Saf..

